# Mesenchymal Stem Cell Therapy for Type 2 Diabetes: Synergistic β‐Cell Regeneration, Immune Modulation, and Exosome‐Mediated Glucose Homeostasis

**DOI:** 10.1155/sci/2759804

**Published:** 2026-06-13

**Authors:** Jia Yang, Yinglong Chen, Huanmei Jing, Chang Liu, Xiaoling Chen, Jiaju Chu, Liyun Chen, Shijie Tang, Fei Yu, Wancong Zhang

**Affiliations:** ^1^ Department of Plastic Surgery and Burn Center, Second Affiliated Hospital, Shantou University Medical College, Shantou, 515051, Guangdong, China, stu.edu.cn; ^2^ Guangdong Provincial Key Laboratory of Marine Biotechnology, Department of Biology, College of Science, Shantou University, Shantou, 515063, Guangdong, China, stu.edu.cn; ^3^ Plastic Surgery Institute of Shantou University Medical College, Shantou, 515051, Guangdong, China; ^4^ Research Center of Translational Medicine, Second Affiliated Hospital of Shantou University Medical College, Shantou, 515051, Guangdong, China, st120.cn

**Keywords:** β-cell repair, stem cells, T2DM, tissue engineering

## Abstract

Type 2 diabetes mellitus (T2DM) is a chronic metabolic disorder characterized by hyperglycemia, insulin resistance, and a progressive decline in pancreatic β‐cell function, leading to impaired glucose regulation. It is often accompanied by severe complications, including cardiovascular diseases, nephropathy, and retinopathy. Conventional treatments, such as sulfonylurea pills, insulin injections, and GLP‐1 receptor agonist injections, provide transient glycemic control but are often costly with unstable efficacy and long‐term side effects. Pancreatic islet transplantation, while effective in some cases, is limited by donor scarcity, graft rejection, and the need for lifelong immunosuppression. Recently, mesenchymal stem cells (MSCs) have emerged as a promising alternative due to their multipotent differentiation potential, immunomodulatory properties, and regenerative capabilities. This review examines the therapeutic roles of MSCs in β‐cell regeneration, inflammation modulation, and tissue repair while also addressing key challenges in clinical translation. However, given the lack of long‐term clinical data, the conclusions presented remain preliminary and should be interpreted with caution. This article, therefore, aims to provide a foundational reference for future research, rather than definitive clinical guidance.

## 1. Introduction

Type 2 diabetes mellitus (T2DM) is a chronic metabolic disorder characterized by insulin deficiency and impaired glucose homeostasis, leading to damage to the eyes, cardiovascular and cerebrovascular vessels, nerves, kidneys, and pancreas that seriously affect quality of life [1]. While pharmacotherapy with metformin, insulin, GLP‐1 receptor agonist, and SLG2 inhibitor reduces hyperglycemia, long‐term use of these medications can cause adverse effects like gastrointestinal discomfort and hypoglycemia [2]. Pancreatic transplantation offers a potential cure, but its widespread use is limited by the scarcity of donor organs, surgical risks, and immune rejection, resulting in high treatment costs and low therapeutic efficacy [3]. These challenges underscore the need for new therapeutic strategies [4]. In recent years, with the rapid advancement of regenerative medicine, stem cell therapy has demonstrated groundbreaking potential in the field of diabetes treatment.

Recent advancements in regenerative medicine have positioned stem cell therapy as a groundbreaking alternative for T2DM treatment [5, 6]. Stem cells, particularly mesenchymal stem cells (MSCs), possess high self‐renewal capacity, plasticity, and immunomodulatory properties, allowing them to differentiate into a variety of cells and tissues, to self‐renew, proliferate, and maintain a functional cell population [7]. MSCs secrete bioactive molecules that promote wound healing and tissue repair, immune modulation, and angiogenesis. MSCs therapy is also less costly and more accessible than islet transplantation [8]. This review systematically elucidates the molecular mechanisms underpinning MSCs’ therapeutic actions while critically addressing current limitations to provide a balanced perspective for future research.

## 2. Characteristics and Biological Basis of MSCs

### 2.1. Characteristics of Stem Cells

Pluripotent stem cells exhibit unlimited self‐renewal capacity and lineage‐specific differentiation plasticity. Stem cells can be modified through genetic engineering techniques, enabling them to provide targeted therapy for specific diseases, thereby offering a new strategy for the treatment of T2DM. They can be classified into embryonic stem cells (ESCs), induced multipotent stem cells (iPSCs), and adult stem cells. Stem cell sources for cell replacement include ESCs [9], iPSCs [10], pancreatic stem cells (PSCs) [11], MSCs [12], hematopoietic stem cells (HSCs) [13], and bone marrow‐derived stem cells (BMSCs) [14]. Among these, MSCs are particularly promising for T2DM treatment because of their wide availability, proliferative capacity, multipotent differentiation potential, and ability to secrete cytokines and extracellular vesicles (EVs).

MSCs exhibit the lowest tumorigenic potential compared to other stem cell types, and the paracrine action of MSCs mediates a wide range of therapeutic effects, which represents a more controllable risk mechanism compared to other stem cell types. Underscoring their enhanced safety profile for therapeutic applications. MSCs have been used in cell‐replacement therapy because of their wide range of sources, proliferative properties, and potential to differentiate into various cell types [6]. MSCs have well‐established safety profiles, manufacturing protocols, and infusion procedures. Their isolation, expansion, and storage processes have been standardized, whereas the production of other stem cell types often requires sophisticated reprogramming techniques and rigorous quality control to prevent mutations, entailing substantially higher costs and more complex manufacturing requirements. Therefore, their therapeutic efficacy stems from their ability to differentiate into target cells, exert paracrine effects that enhance endogenous cell activity and survival, and provide desirable immunomodulatory effects [3, 4]. These key core mechanisms collectively underpin and propel the clinical translation of stem cell therapies [15].

### 2.2. Pathogenesis of T2DM and Therapeutic Targets of MSCs

#### 2.2.1. Pathogenesis of T2DM

The pathogenesis of T2DM is driven by two interrelated defects: impaired insulin secretion from pancreatic β‐cells and diminished insulin sensitivity in peripheral tissues [16]. In the early stages, β‐cells compensate for insulin resistance by increasing insulin output, but over time, chronic metabolic stress—including glucotoxicity, lipotoxicity, and oxidative stress—leads to β‐cell dysfunction, dedifferentiation, and apoptosis 17–19]. Key molecular pathways involved include the PI3K/Akt cascade, which mediates insulin signaling; recent evidence suggests that elevated interferon‐stimulated gene 15 (ISG15) expression in T2DM patients can disrupt the PI3K/Akt signaling axis, thereby aggravating tissue oxidative stress [20]. Additionally, endoplasmic reticulum stress and mitochondrial dysfunction further compromise β‐cell survival and function.

#### 2.2.2. Chronic Inflammation and Immune Dysregulation

**Figure 1:**
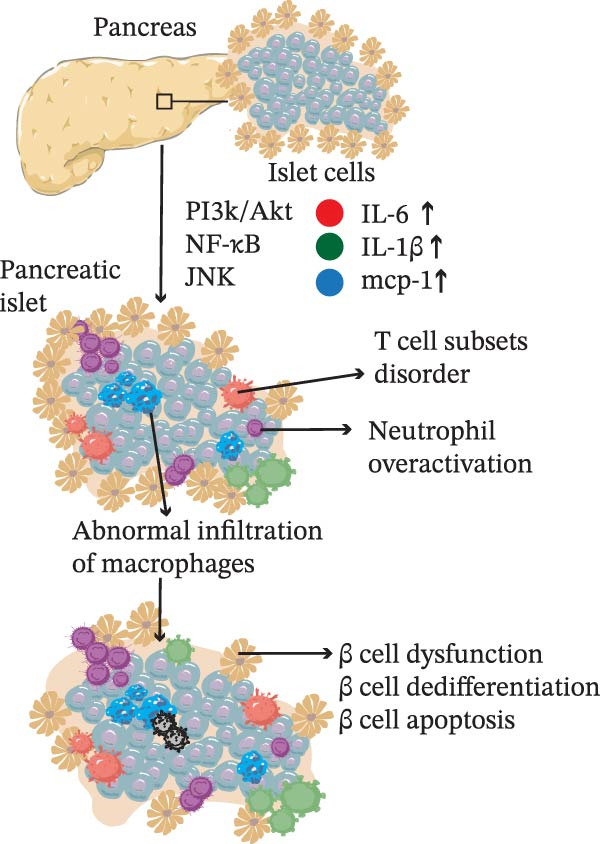
Mechanism of islet cell dysfunction and its complications in T2DM. *Source:* This image is an original creation by the author.

The chronic inflammatory microenvironment of T2DM activates the PI3K/Akt, NF‐κB, and JNK pathways, disrupting immune homeostasis, and elevates related factors such as TNF‐α, interleukin (IL)‐6, IL‐1β, and monocyte chemoattractant protein‐1 (MCP‐1), accelerating β‐cell dysfunction and apoptosis [20] (Figure 1). Key manifestations include abnormal macrophage infiltration, neutrophil hyperactivation, and dysregulated T‐cell subset polarization. This abnormal immune response further intensifies local and systemic inflammatory responses, ultimately inflicting irreversible damage on pancreatic β‐cells [20]. A comprehensive understanding of these interconnected pathogenic mechanisms is crucial for developing targeted therapies, such as MSC‐based approaches for reversing β‐cell dysfunction and modulating inflammation [17–19].

#### 2.2.3. Pathophysiological Basis of MSC Therapy for T2DM

Given the multifactorial pathogenesis of T2DM, an ideal therapeutic strategy must simultaneously address β‐cell insufficiency, insulin resistance, and chronic inflammation. MSCs fulfill these criteria through three core mechanisms: (i) their capacity to differentiate into insulin‐producing cells (IPCs) or to stimulate endogenous β‐cell regeneration [9–12]; (ii) their potent immunomodulatory effects, which re‐establish immune homeostasis by promoting regulatory T cell (Treg) induction and skewing macrophages toward an anti‐inflammatory M2 phenotype [17–19]; and (iii) their paracrine release of EVs and trophic factors, which enhance insulin sensitivity, inhibit apoptosis, and promote vascular and tissue regeneration [13–15]. By directly targeting the root causes of T2DM, MSCs demonstrate transformative therapeutic potential.

## 3. Biological Characteristics of MSCs

### 3.1. Differentiation Potential

Direct differentiation of MSCs into IPCs represents a conceptually straightforward approach to restore β‐cell mass. MSCs pluripotency enables directed differentiation of human islet MSCs and bone marrow‐derived MSCs into IPCs through lineage‐specific induction protocols employing retinoic acid, activin, and GLP‐1 [21]. Multiple studies have confirmed the feasibility of this approach across different MSC sources. For example, goat adipose tissue‐derived MSCs were successfully induced into IPCs using a combination of glucose, nicotinamide, activin A, exendin‐4, pentagastrin, retinoic acid, and β‐mercaptoethanol, with the resulting cells expressing pancreatic transcription factors such as *Pdx1*, Ngn3, and Glut2 [22]. Another study demonstrated that human adipose tissue‐derived MSCs could be directed toward an insulin‐producing phenotype within approximately 20 days using activin A, CHIR99021, wortmannin, trichostatin‐A, GLP‐1, exendin‐4, nicotinamide, and PRDX6, ultimately forming islet‐like clusters co‐expressing insulin and C‐peptide [23]. Human gingival MSCs have also been shown to generate IPC clusters via a three‐step serum‐free protocol involving activin A, taurine, GLP‐1, and nicotinamide, with the resulting cells exhibiting glucose‐responsive insulin secretion and expression of *Pdx1* and glucagon [24]. In rat models, adipose‐derived MSCs have been successfully differentiated into IPCs using a high‐glucose medium supplemented with β‐mercaptoethanol, nicotinamide, and exendin‐4, with the differentiated cells demonstrating key β‐cell marker expression and functional insulin secretion. Additionally, a study has established a novel method employing activin A, retinoic acid, and nicotinamide for efficient differentiation of umbilical cord MSCs (UC‐MSCs) into functional pancreatic β‐cells [22]. MSCs can be induced to differentiate into IPCs that not only express insulin and *Pdx1* but also exhibit glucose‐dependent regulation of insulin secretion [23, 24]. Stem cell reprogramming involves incorporating exogenous target genes into MSCs for specific roles in disease treatment. For example, *Pax4*, which encodes a transcription factor involved in β‐cell development and function, synergistically acts with the transcription factors, *Pdx1*, Ngn3, and MafA, to reprogram human MSCs (hUC‐MSCs) to differentiate into functional β‐cells. Research using canine diabetes as a model has demonstrated the potential of reprogramming to differentiate adipocyte‐derived MSCs (ADMSCs) into β‐cells for T2D treatment. The researchers successfully reprogrammed ADMSCs into cells with β‐cell characteristics through a six‐gene combination (*Pbx1*, *Rfx3*, *Pdx1*, *Ngn3*, *Pax4*, and *MafA*) [25, 26].

### 3.2. Paracrine Function and Exosomes

EVs mediate intercellular signaling to facilitate diabetic wound repair [27]. MSC‐derived exosomes loaded with miR‐26a–5p have demonstrated protective effects in diabetic nephropathy (DN) in a diabetic rat model by suppressing oxidative stress, excessive inflammatory responses, and macrophage infiltration [28]; other MSC‐EVs afforded protection against cerebrovascular injury by regulating the AMPK and JAK2/STAT3/NF‐κB pathways, which could be effective in targeting hyperglycemia‐induced cerebrovascular pathogenesis in diabetes [29].

### 3.3. Immunomodulatory Properties

Additionally, paracrine factors from MSCs suppressed xenotransplant rejection, highlighting their immunoregulatory functions [30]. hUC‐MSCs mediate lymphocyte immunomodulation through cell contact‐dependent mechanisms and paracrine cytokine regulation, suggesting that direct cellular contact and suppression of cytokine secretion from human peripheral blood lymphocytes may be involved in the immunomodulatory effect of hUC‐MSCs [31]. In addition, hUC‐MSCs restored the Th17/Treg balance and maintain the homeostasis of the IL‐17/TGF‐β network through epigenetic reprogramming of the RORγt/Foxp3 axis, ultimately reducing inflammation [32].

These findings establish MSC‐based therapies as a viable strategy for T2DM treatment, leveraging mechanisms including stem cell pluripotency, reprogramming capacity, and paracrine actions. The following section will delve into these mechanisms in greater detail, specifically in the context of treating T2DM.

## 4. Mechanisms of MSCs in T2DM Therapy

**Figure 2:**
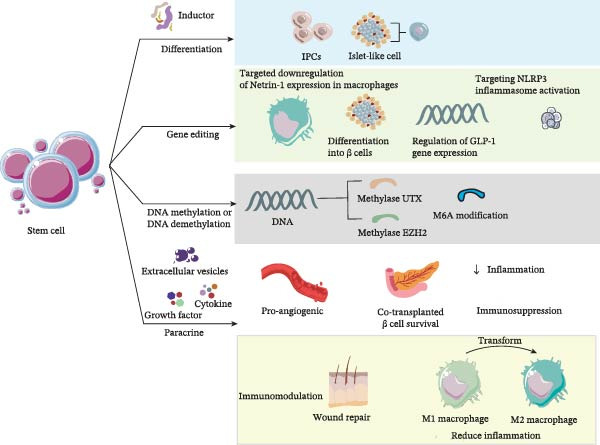
Therapeutic mechanism of mesenchymal stem cells in T2DM. *Source:* This image is an original creation by the author.

MSCs can directly differentiate into IPCs to restore β‐cell mass. The mechanisms of stem cells’ effects on T2DM primarily involve lateral differentiation, cell fusion, DNA methylation, and paracrine signaling [33, 34] (Figure 2). Stem cells are able to differentiate into insulin‐secreting β‐like cells [35] and possess paracrine effects that can heighten endogenous β‐cell activity, safeguard the survival of co‐transplanted β‐cells, reduce IR, and restore normal immune responses [36, 37].

### 4.1. Inducing Differentiation

Stem cells can be induced to differentiate into β‐like cells by incubation with different factors. Previous research has shown that adding GABA, BMP7, and activin to the growth medium induces adult rat pancreatic ductal epithelial stem cells (PDESCs) to differentiate into β‐like cells in vitro [38], with the capacity for producing insulin [38, 39]. A study successfully generated insulin and C‐peptide co‐expressing β‐like cells from human amniotic epithelial cells (hAECs) by sequentially modulating activin/WNT, KGF/TGF‐β, retinoic acid/hedgehog, and EGF/Noggin signaling through a protocol that mimics in vivo pancreatic development [39]. Optimization of cell culture conditions facilitates intercellular interactions and activates intracellular signaling cascades, thereby enabling the differentiation of MSCs into insulin‐secreting β‐like cells. In an experiment, hAT‐MSCs were successfully differentiated into IPCs and subsequently transplanted into streptozotocin (STZ)‐induced diabetic humanized NOG‐EXL mice. The results demonstrated that the transplanted IPCs significantly reduced blood glucose levels without eliciting immune responses [40].

### 4.2. Epigenetic Regulation and Gene Editing

Epigenetics plays an important role in the occurrence and development of diabetes and also serves as a therapeutic target [41]. Histone demethylase UTX expression is significantly upregulated in renal tissues of diabetic mouse models and patients with DN, with predominant localization in glomerular mesangial cells and renal tubular epithelial cells; decreasing UTX demethylase activity improved renal function and attenuated hyperglycemia‐induced renal pathology, inflammation, and DNA damage, suggesting that UTX contributes to these detrimental effects [42]. Microenvironmental changes resulting from diabetes cause shifts in stem cell methylation levels. A recent study showed that BMSC transplantation increased the expression of histone methyltransferase, EZH2, and decreased the expression of histone demethylase, KDM6B, in diabetic individuals [43]. This finding suggests a novel approach for examining the epigenetic mechanisms involved in diabetic bone abnormalities by altering DNA methylation levels through gene editing systems to provide more precise treatment of T2DM conditions. In addition to direct cell‐based therapies, MSCs offer a cell‐free therapeutic strategy for diabetes through the epigenetic engineering of their secreted exosomes, with the underlying mechanism believed to be closely associated with the RNA molecules encapsulated within these exosomess. Notably, genetically engineered MSC exosomes, through the introduction of m6A modifications, can achieve the epigenetic silencing of the myostatin (MSTN) gene, thereby exhibiting significant potential for attenuating the progression of DN [44].

The CRISPR/Cas9 and CRISPR–Cpf1 systems offer new technologies for diabetes treatment by several methods including stem cell induction into β‐cells, gene editing for reprogramming into β‐cells, and target gene modification 45–47]. The CRISPR/Cas9 gene editing platform enables multipronged intervention in diabetes pathogenesis through several coordinated mechanisms: first, by directing the differentiation of hESCs into pancreatic β‐cells to expand the functional β‐cell mass and restore insulin secretion; second, by precisely modulating GLP‐1 gene expression to further enhance insulin secretory capacity; third, by targeting the NLRP3 inflammasome to suppress its activation, thereby attenuating inflammatory responses and ameliorating insulin resistance; and fourth, by overexpressing hepatocyte growth factor (HGF) and other approaches to inhibit programmed cell death in stem cells. On the other hand, transduction of MSCs with the ERBB4 gene enhanced their anti‐apoptotic capacity via activation of the PI3K/AKT signaling pathway [46]. Fifth, by targeting downregulation of Netrin‐1 expression in macrophages to mitigate their infiltration into adipose tissue, consequently ameliorating chronic inflammation and insulin resistance associated with diabetes [45, 47]. The CRISPR/Cas9 gene editing system has also been used for studying *HLADR-DQ*, *HLA-B*, *HLA-A* (key factors of immune regulation), and *INS* (insulin secretion) as potential gene targets in the treatment of diabetes. *TCF7L2* (key factors in blood glucose regulation), *IGF2BP2* (involved in insulin signaling and metabolism), IRS1 (key mediator of insulin receptor signaling), and KCNQ1 (affecting insulin secretion of pancreatic β‐cells) have also been edited in T2DM research [9, 48]. Precise genetic modification of stem cells using advanced gene editing techniques significantly enhances their therapeutic efficacy and targeting in treating T2DM. For example, gene editing to induce high expression of HGF or overexpression of specific chemokines in stem cells greatly augments their homing capacity, enabling directed migration to damaged tissues [48].

### 4.3. Paracrine Effects and Exosome‐Mediated Repair

Stem cell paracrine activity constitutes an effective means of diabetes therapy using EVs released by MSCs. Compared with iPSCs, the tumorigenic risk associated with undifferentiated iPSCs represents a major hurdle in their clinical translation. The reprogramming process of iPSCs may introduce epigenetic abnormalities and genomic instability [49]. In contrast, MSCs exhibit more stable biological properties and have a relatively well‐established quality control system. Rather than direct differentiation, the paracrine function of MSCs plays a more critical role in the treatment of T2DM. Through the secretion of abundant trophic factors, cytokines, and exosomes, MSCs exert potent anti‐inflammatory, immunosuppressive, anti‐apoptotic, and pro‐angiogenic effects. Specifically, MSC‐EVs can carry many types of proteins, DNA, mRNA, and signal transduction molecules [50]. In the field of islet transplantation for T2DM, these cells can interact with target cells through internalization, ligand‐receptor interactions, cytokine secretion, and gene regulation to increase the survival and promote normal functioning of co‐transplanted and endogenous β‐cells [50].

### 4.4. Immune Modulation and Inflammation Control

In the field of chronic wound therapy for T2DM, human amniotic MSC exosomes (hAMSC‐exos) carrying OIP5‐AS1 have been shown to improve human umbilical vein endothelial cell (hUVEC) function after high‐glucose damage by targeting miR‐29a‐3p to upregulate the expression of SIRT1 and activate the PI3K/Akt pathway. This promotes the proliferation, migration, and vessel formation of hUVECs under high glucose conditions, which is beneficial for diabetic wound healing [51]. The use of exosomes from apical papilla stem cells (SCAPs‐exos) for the treatment of skin defects caused by diabetes in a model where skin defects were created in the facial region surrounding the anterior teeth revealed that SCAP‐exos enhanced the healing of skin defects in diabetic mice by increasing the level of treg cells [52]. Additionally, the possible mechanism involved the activation of the SMAD2/3 pathway through the upregulation of TGF‐βR1 expression. The demethylation of the Foxp3 gene locus by TET2 facilitated the positive and stable expression of Foxp3, promoting the transformation of Tregs and their functional stabilization [52]. Within the paracrine actions of MSCs, the impact of efferocytosis apoptotic cells on macrophage function—particularly their polarization—represents another key mechanism in the treatment of T2DM. Studies have shown that apoptotic MSCs, upon efferocytosis by macrophages, promote the polarization of macrophages toward the anti‐inflammatory M2 phenotype. This mechanism allows MSCs to sustain their anti‐inflammatory activity even after undergoing apoptosis in vivo, thereby mitigating the inflammation associated with T2DM [50].

In summary, MSCs exert their core therapeutic effects in T2DM through a synergistic multimechanism network: they achieve functional reconstitution of β‐cells via directed differentiation, restore insulin secretion, and precisely regulate key targets through epigenetic and gene‐editing approaches to improve insulin resistance and metabolic homeostasis, and mediate extensive systemic repair via paracrine/exosomal signaling, thereby orchestrating a coordinated and enhanced therapeutic outcome. Thereby achieving a synergistic effect on blood glucose control and complication management.

## 5. Current Research on MSC Therapy for T2DM

MSCs can exhibit remarkable therapeutic effects in T2DM through multiple mechanisms, including differentiation into functional IPCs, regulation of immune homeostasis, promotion of tissue repair, and exertion of paracrine effects.

### 5.1. Preclinical Evidence in Animal Models

Animal models, commonly including mice fed a high‐fat diet (HFD) and injected with STZ have been crucial for evaluating MSC therapy. This model mimics the pathological features of human T2DM by inducing insulin resistance through HFD feeding and pancreatic β‐cell destruction via intraperitoneal STZ injection. Current research has shown that MSCs, administered through direct infusion, transplantation of induced β‐like cells (IPCs), or exosomal therapies, can alleviate T2DM‐related complications such as chronic wounds, liver and kidney injury, and pancreatic islet damage [35, 37, 38] (Table 1).

**Table 1:** Treatment strategies for MSCs and mechanisms of action.

Treatment strategies	Mechanisms of action
MSC infusion	Immune regulation: Inhibits pro‐inflammatory T cells, promotes generation of Treg cells, modulates immune responses, reduces oxidative stress [34]
	Ameliorates cardiac function in T2DM rats by suppressing JAK2/STAT3/iNOS pathway [53]
	Modulating immune responses and promoting β‐cell regeneration, insulin secretion is improved, diabetic complications are prevented [54]
	Regulates blood glucose levels, protects islet cells, promotes vascularization [55]
Induced differentiation of MSCs into IPCs	Ameliorates hyperglycemia in diabetic rats, restores insulin/C‐peptide, upregulates the expression of genes related to β‐cell function [56] Core inducing factors synergistically drive MSC differentiation into IPCs [57] CRISPR activates *Pdx1*/*NeuroD1*/*MafA* to convert MSCs into IPCs [58] Pancreatic MSC‐ex boosts β‐cell regeneration [59]
Exosomes from MSCs	MSC‐exos mitigate T2DM by boosting blood flow, modulating immunity, and protecting β‐cells [51, 52] Reduce hyperglycemia and boost insulin secretion in T2DM rats by reducing insulin resistance and protecting β‐cells [60]. MSCs‐derived apoptotic vesicles are efficiently engulfed by liver macrophages, which subsequently polarize toward the anti‐inflammatory M2 phenotype [50]

MSCs infusion modulates immune responses by suppressing pro‐inflammatory T cells and promoting regulatory T cells, thereby reducing oxidative stress, systemic inflammation, and renal and hepatic injury. Human adipose‐derived MSCs (hASCs) were investigated for diabetic chronic wound repair. Evaluation using a full‐thickness skin defect model on the dorsum of diabetic mice demonstrated that hASCs significantly accelerated wound closure and effectively promoted angiogenesis [34]. Studies have demonstrated that MSC transplantation significantly improved cardiac function [53]. This study established a T2DM rat model using a HFD combined with STZ and divided the animals into a control group, a model group, and a bone marrow‐derived MSC (BM‐MSC) treatment group (intravenous injection of 2 × 10^6^ cells). The results showed that 2 weeks after MSC treatment, insulin resistance, dyslipidemia, and cardiac injury markers were significantly improved, while the cardiac JAK2/STAT3/iNOS and apoptotic pathways were downregulated, and left ventricular function was restored. The conclusion suggests that MSCs alleviate diabetic cardiomyopathy by inhibiting these pathways [53]. Study by Rhew et al. [54] demonstrated the efficacy and safety of intravenous infusion of allogeneic canine adipose tissue‐derived MSCs (cAT‐MSCs) in four dogs with insulin‐dependent diabetes mellitus (IDDM) that had received insulin therapy for more than 1 year. After three or 5 months of infusions, C‐peptide levels increased by 5%–15% in three dogs, fructosamine and HbA1c improved in two dogs, hyperlipidemia was alleviated in two dogs, and body weight and appetite improved in two dogs; only one dog experienced a mild injection site reaction. The study suggests that cAT‐MSCs may improve insulin secretory capacity and help prevent complications in dogs with IDDM. A study by Yamashita et al. [55] demonstrated that subcutaneous transplantation of sheets of MSCs effectively regulated blood glucose levels in diabetic pigs and achieved long‐term glycemic stability. MSCs not only protected islet cells but also promoted vascularization, providing an effective platform for islet transplantation. In this study, ADSCs were isolated from the inguinal subcutaneous fat of young pigs to prepare cell sheets, and islets isolated from adult pig pancreata were seeded onto the ADSC sheets for co‐culture. Diabetes was induced in pigs by total pancreatectomy, and the islet/ADSC sheets were then transplanted subcutaneously into the waist region of the model pigs. The experimental groups included a normal control group, a model group, and an islet/ADSC sheet transplantation treatment group (*n* = 2), with a self‐control consisting of graft resection performed 14 days post‐transplantation. Another prominent research direction involved in vitro differentiation of MSCs into IPCs for transplantation [39].

A study by Ghahremani Piraghaj et al. [37] found that efferocytosis of apoptotic adipose‐derived MSCs polarizes macrophages to an M2 anti‐inflammatory phenotype in C57BL/6 mice, as evidenced by a suppressed TNF‐α/NO response and enhanced IL‐10/arginase activity. This establishes that MSCs maintain immunomodulatory function post‐apoptosis, providing a key experimental basis for optimizing T2DM cell therapies [37]. In a HFD‐induced T2DM mouse model, Zheng et al. [50] demonstrated that intravenously administered MSCs‐derived apoptotic vesicles (apoVs) are efficiently efferocytosed by liver macrophages via the surface receptor calreticulin (CRT). This efferocytosis prompted macrophage polarization toward the anti‐inflammatory M2 phenotype, which reduced monocyte infiltration and pro‐inflammatory cytokine secretion. Consequently, it restored hepatic immune homeostasis and significantly improved glucose tolerance, insulin resistance, and hepatic steatosis. These findings confirm the novel potential of apoVs as a cell‐free therapeutic agent for T2DM [50]. In the animal experiment section, the authors established a normal diet control group, an HFD model group, and an apoV treatment group. Additionally, they further set up apoV intervention groups with CRT blockade or knockdown using a CRT‐neutralizing antibody or siRNA, respectively, and also compared apoptosis‐deficient Fas mutant mice with wild‐type mice.

The induction efficiency was enhanced with small‐molecule compounds or combinations of insulin‐like growth factor and epidermal growth factor. Genetic modification has also been shown to significantly improve the induction efficiency. These induced IPCs can respond to glucose fluctuations and secrete insulin, thereby mimicking the function of β‐cells in vivo. In animal models, the transplantation of differentiated IPCs has been shown to significantly improve glycemic control and reduce the need for exogenous insulin. The study by Hosseini et al. [57] demonstrated that the GLP‐1 receptor agonist, Exendin‐4, and nicotinamide, as key induction factors, could induce the differentiation of MSCs into IPCs. Transplantation of the induced IPCs beneath the renal capsule of diabetic nude mice ameliorates hyperglycemia [57]. Aglan et al. [56] demonstrated that IPCs derived from MSCs through specific induction protocols exhibited significant insulin secretion capacity and therapeutic potential in vivo. These cells were able to reduce hyperglycemia in diabetic rats, restore insulin and C‐peptide levels, and upregulate the expression of genes related to β‐cell function in the pancreas.

The CRISPR–Cas9 system can precisely edit the genome of MSCs by activating the expression of specific transcription factors, such as *Pdx1*, *NeuroD1*, and *MafA*, thereby inducing the differentiation of MSCs into IPCs [58]. The treatment elicited significant therapeutic effects in STZ‐induced diabetic mice, including restored blood glucose levels, improved insulin secretion, and established immune tolerance [58]. Genetically modified MSCs expressing gastrin have shown significant potential in protecting β‐cells and improving islet function in diabetic mice [61]. Pancreas‐derived MSCs can express pancreatic progenitor cell markers that may promote β‐cell regeneration via exosomes [59], and MSC‐derived exosomes contain bioactive factors including cytokines (IL‐10 and TGF‐β), chemokines (SDF‐1), and growth factors (VEGF, HGF, and IGF‐1) that promote angiogenesis, modulate immune responses, reduce inflammation, and protect β‐cells in T2DM mice [51, 52]. Sun et al. [60] demonstrated that exosomes derived from MSCs significantly lowered blood glucose levels and increased insulin secretion in T2DM rats by reversing peripheral insulin resistance and alleviating β‐cell destruction. These studies collectively indicate that MSCs possess substantial potential for the treatment of T2DM.

### 5.2. Clinical Advances

An increasing number of clinical studies have demonstrated that MSC therapy for T2DM not only effectively reduces blood glucose levels but also alleviates chronic inflammation in patients, fundamentally alleviating the disease pathology and improving the quality of life. Evidence proves that MSCs treatment can promote regeneration and functional recovery of pancreatic islet cells, thereby reducing patient dependence on insulin (Table 2). Studies with follow‐ups at 6 and 12 months have shown that patients treated with MSCs experienced a significant reduction in insulin usage. Fasting blood glucose and glycated hemoglobin levels were markedly decreased throughout the follow‐up period, while C‐peptide levels were significantly increased, indicating the restoration of normal insulin regulation and secretion by pancreatic β‐cells [62]. Studies have shown that MSC therapy not only reduces hyperglycemia by promoting the regeneration of functioning pancreatic β‐cells but also reduces the risk of diabetic complications through multiple mechanisms. For example, MSCs can modulate immune responses by increasing the population of regulatory T cells, thereby controlling inflammatory reactions and mitigating the damage caused by hyperglycemia [63]. At 1 month after MSC transplantation, patients exhibited a significant increase in fasting C‐peptide levels, peak C‐peptide release, and area under the C‐peptide curve [63]; these improvements were consistently observed throughout the follow‐up period. as evidenced in clinical studies. Data from a retrospective study showed that in patients with type 2 diabetes who received intravenous infusion of UC‐MSCs, estimated glomerular filtration rate (eGFR) was significantly increased and blood urea nitrogen (BUN) levels were significantly decreased at both 6‐ and 12‐month follow‐ups. These findings provide direct evidence supporting the renoprotective effects of MSCs [64]. These findings provide preliminary clinical evidence supporting the use of MSCs in the treatment of T2DM, highlighting their immense potential and broad prospects in alleviating diabetic pathology, promoting islet cell regeneration, modulating the immune system, and reducing complications. Zang et al. [65] conducted a study in which 45 patients were randomly assigned to receive MSC therapy. After 48 weeks of treatment, 20% of the patients in the MSC treatment group achieved a glycated hemoglobin (HbA1c) level below 7.0% and a reduction in daily insulin usage by at least 50%. The MSC treatment group also showed a significant increase in the glucose infusion rate (GIR), indicating an improvement in insulin resistance. A study by Mikłosz et al. [66] of a novel MSCs therapeutic strategy for diabetes demonstrated great potential in improving glycemic control, reducing insulin requirements, and promoting β‐cell regeneration. Wu et al. [67] conducted an 8‐year randomized controlled study in patients with T2DM who received combined bone marrow MSCs and mononuclear cells. The treatment significantly increased the C‐peptide area under the curve by 50.6% at 1 year and markedly reduced the incidence of macrovascular complications and diabetic peripheral neuropathy, demonstrating the long‐term benefits of MSC‐based therapy in improving metabolic control and reducing chronic complications. The report by Madhoun et al. [68] revealed another potential application of MSCs in diabetes treatment for improving islet blood supply through the promotion of angiogenesis.

**Table 2:** Current application and progress of MSCs in clinical treatment of T2DM.

Key findings	The impact on patients
MSCs therapy promotes pancreatic islet cell regeneration and functional recovery [62]	Fasting blood glucose decreased, C‐peptide level increased, HbA1c level increased
Promote tissue repair, regulatory T cell abundance, modulates inflammatory responses [63]	Shorten the healing time of diabetic foot, increase granulation tissue formation, promote epithelialization, improve the regeneration of skin appendages (hair follicles), promote angiogenesis, and reduce local inflammatory factors
Promoting islet cell regeneration and modulating the immune system [64]	Fasting C‐peptide increased significantly and remained elevated through 24 months, with partial restoration of islet function. HbA1c decreased markedly and was maintained at lower levels during the 24‐month period, with no immune rejection observed
Significant reduction in fasting blood glucose and glycated hemoglobin (HbA1c) levels [65, 66]	Glycated hemoglobin (HbA1c) decreased and 13.5% of patients achieved insulin discontinuation within 8–24 weeks, with a mean duration of 37.2 ± 15.2 weeks. This was accompanied by reduced blood glucose levels and a transient increase in glucagon‐stimulated C‐peptide, which peaked and subsequently declined after 20 weeks, along with significantly improved insulin sensitivity

Notwithstanding the preliminary clinical evidence supporting MSC‐based therapy for T2DM provided by the aforementioned studies, current research remains constrained by several limitations, including small sample sizes, inconsistent follow‐up durations, and substantial heterogeneity in baseline patient characteristics, which collectively contribute to the limited strength of the available evidence. Considerable variability exists in MSC sources, manufacturing protocols, routes of administration, and dosages across studies, precluding meaningful cross‐trial comparisons and hindering the formation of a clinical consensus. Furthermore, existing studies generally lack systematic long‐term follow‐up data on the durability of therapeutic efficacy and the long‐term safety profile of MSC administration, rendering the evidence insufficient to support its widespread adoption as a standard‐of‐care treatment. Urgently needed are large‐scale, well‐designed studies, alongside the establishment of standardized MSC manufacturing and quality control frameworks, to definitively establish their efficacy, safety profile, and optimal clinical application protocols.

### 5.3. Genetic Engineering and Chimeric Antigen Receptor (CAR)‐MSC Strategies

**Figure 3:**
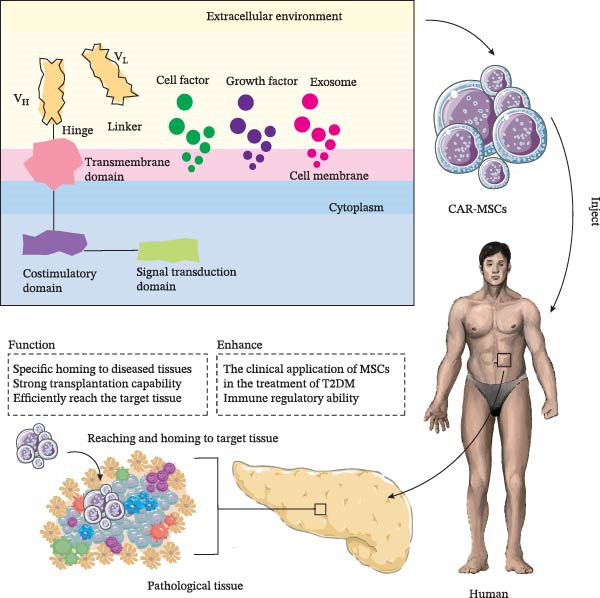
Structure and function of CAR‐MSCs used in T2DM therapy. *Source:* This image is an original creation by the author.

The CAR is a genetically engineered cell receptor that enables modified cells to specifically recognize and attack cells expressing a particular antigen [69]. The CAR consists of two components: an extracellular portion with a single‐chain variable fragment (scFv) that confers antigen specificity and an intracellular portion containing signaling domains that activate specific cellular functions (Figure 3). While initially developed for cancer immunotherapy, CAR technology has emerged as a promising approach for T2DM treatment by enhancing the therapeutic potential of MSCs [70].

CAR‐MSCs possess strong transplantation potential and tropism capabilities, enabling them to efficiently reach target tissues [71]. CAR‐MSCs represent an emerging cell therapy strategy that combines CAR technology with MSCs, endowing MSCs with the ability to specifically home to diseased cells. This approach improves MSCs therapeutic efficacy and immunomodulatory functions in T2DM treatment [72–74]. In recent years, CAR‐like MSCs have been introduced into the treatment of T2DM [75], and the key focus of CAR‐Like MSCs lies in achieving CAR‐like targeting effects without necessarily relying on the classical CAR structure. This can be accomplished through chemical modifications, physical adsorption, or combination with other cytokines to mimic CAR functionality and does not necessarily involve genetic editing techniques. Currently, research on CAR‐Like MSCs is still at a very early laboratory stage, but some preliminary results have been achieved [76]. For example, a new type of CAR‐like MSC for T2DM treatment was successfully developed in China, which has been granted international patents and received official approval for T2DM clinical trials, offering patients a new therapeutic option [76, 77]. Nevertheless, these advances are still at an early exploratory stage, and there remains a long way to go before they become a routine treatment for T2DM. By integrating genetic engineering with exosome technologies, CAR‐MSCs have been shown to modulate immune responses and restore pancreatic islet function, offering new therapeutic strategies for T2DM patients [78, 79]. The incorporation of CAR technology into MSCs confers antigen‐targeting capability while preserving their inherent immunomodulatory, tissue repair, and homing properties [70]. Clinical research on CAR‐MSCs in humans remains at an early stage, with no large‐scale registered clinical trials or published clinical data available to date. Their application in T2DM treatment is similarly nascent. Nevertheless, several studies have confirmed the efficacy of CAR‐MSCs across multiple disease models. CAR‐MSCs engineered to target various antigens—such as GD2, CD33, NKG2D, and E‐cadherin—and armed with payloads including TRAIL, bispecific antibodies, or cytokines have shown significant disease suppression and extended survival in models of glioblastoma, Ewing sarcoma, acute myeloid leukemia (AML), non‐small cell lung cancer (NSCLC), and graft‐versus‐host disease (GvHD), with favorable safety profiles [78]. The Mayo Clinic [79] is currently designing the world’s first Phase I clinical trial of CAR‐MSCs for human use, which will utilize adipose‐derived CAR‐MSCs targeting E‐cadherin with CD28ζ signaling domains to treat steroid‐refractory acute GvHD. Leveraging the dual advantages of targeted delivery and immunomodulation offered by CAR‐MSCs, this approach holds promise to overcome the limitations of conventional T2DM therapies, enabling a strategic transition from “inflammatory amelioration” and “glycemic control” to “islet restoration.” However, it must be clearly recognized that this vision currently remains at the conceptual and preclinical exploratory stage and is still in its infancy in the field of T2DM therapy, and there are many challenges to overcome before practical application.

### 5.4. Tissue Engineering Strategies

**Figure 4:**
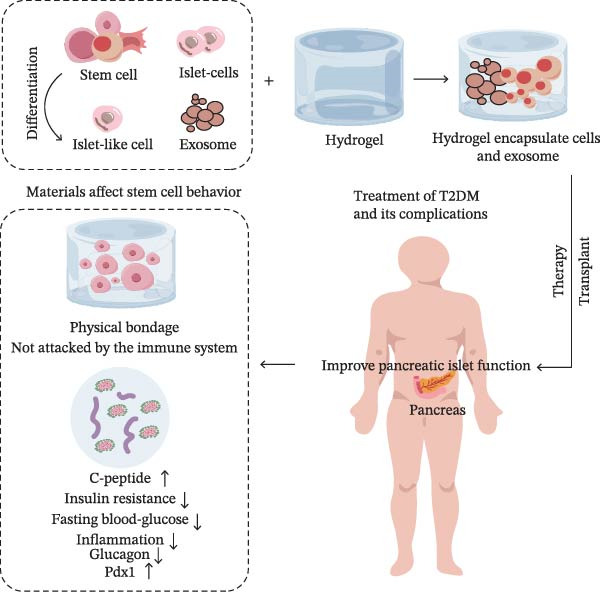
Treatment of T2DM with stem cells or exosomes encapsulated within hydrogels. *Source:* This image is an original creation by the author.

While MSCs therapy shows much promise, challenges such as aberrant differentiation, tumorigenicity, and limited retention at target sites necessitate the use of carriers that physically support MSCs and provide a favorable microenvironment (Figure 4). Two‐dimensional (2D)‐cultured stem cells do not adequately mimic the in vivo pancreas environment. However, human‐induced pluripotent stem cells (hiPSCs) cultured on a 3D scaffold have been shown to express significantly higher levels of T2DM‐relevant biomarkers (*Pdx1*, glucagon, and *Glut2*) [80], with critical implications for the use of bioscaffolds as MSCs supports in the treatment of T2DM.

Culturing MSCs on hydrogel materials that mimic the extracellular matrix (ECM) has been found promising for T2DM therapy [38, 52, 81]. Hydrogels provide a stable, biocompatible 3D structure able to retain a relatively large amount of water, which has stimulated much interest in hydrogels for T2DM therapy, particularly in promoting pancreatic islet differentiation and wound healing [82–85]. Given the scarcity of islets for transplantation, hydrogels are being explored for their potential in β‐cell generation and transplantation [86, 87]. With advancing research, the application of exosomes in the diabetes treatment has also garnered increasing attention. The integration of exosomes with hydrogels and other tissue engineering materials for diabetes management has gained momentum, along with the elucidation of their molecular mechanisms in diabetes therapy, and is expected to become a major focal point in future research [88, 89].

Hydrogel networks can be synthesized from natural macromolecules, such as collagen, fibrin, hyaluronic acid and alginate, or from synthetic polymers like polyacrylamide (PAM) and polyethylene glycol (PEG). Natural‐derived hydrogels have been used in wound repair, islet transplantation, and islet organoid engineering [90, 91]. Alginate‐based MSCs delivery systems significantly enhance cell viability and paracrine activity while demonstrating clinically relevant efficacy in T2DM [92]. Chitosan hydrogels, loaded with BMSCs‐exos, have been proven effective in treating chronic diabetic wounds [93]. Pancreatic islet cells derived from stem cells and endothelial cells were enmeshed in the hydrogel to form three‐dimensional aggregates, and the resulting islet‐like organs successfully expressed β‐cell and other endocrine markers [94].

Synthetic hydrogels, composed of materials like polyethylene oxide (PEO), PEG, polyvinyl alcohol (PVA), and PAM, offer therapeutic strategies in wound regeneration, endogenous islet enhancement, controlled stem cell delivery, and de novo islet organoid engineering. A hybrid injectable hydrogel composed of hyperbranched PEG loaded with ADSCs could potentially expedite the diabetic wound‐healing process [95]. Nanomaterials incorporated into PVA‐alginate hydrogels embedded with stem cell exosomes were also found to aid in the wound healing process [96]. Electrospun PVA chitosan dressings possess ROS scavenging and antimicrobial properties to enhance healing [97]. Ligand‐functionalized PEG hydrogels loaded with MSCs load ECM‐derived cell adhesion ligands and GLP‐1 can significantly improve pancreatic islet function [98, 99]. Thermoresponsive poly (N‐isopropylacrylamide) hydrogels can be used for culturing adipose‐derived MSC spheroids [100]. Temperature‐sensitive hydrogels have been shown to be efficient carriers of cultured rabbit AD‐MSCs [101, 102]. Current research in organoid engineering confirms the feasibility of using 3D scaffolds made of poly‐L‐lactic acid and PVA [103]. This breakthrough establishes a foundational framework for leveraging *de novo*‐engineered islet organoids in the treatment of T2DM.

Hydrogels can be synthesized from PEG‐modified natural polymers such as heparin, dextran, hyaluronic acid, fibrinogen and albumin, and natural polymers like collagen, chitosan, and alginate that have been modified with p (NIPAAm) [95, 96]. These scaffolds can be used for successful encapsulation and delivery of MSCs and used in diabetes therapy. A biomimetic hydrogel comprising a maleimide‐modified hyaluronic acid with a collagen mimetic peptide, 8‐CG‐RGDS, for islet organoids [104]. RGD‐modified self‐assembled D‐peptide hydrogels improved the therapeutic effects of MSCs on hindlimb ischemia by promoting angiogenesis [105]. Modified natural polymer‐based hydrogels show therapeutic potential for chronic T2DM wound management. Hydrogels synthesized from insulin‐modified decellularized adipose tissue/tremella polysaccharides loaded with ADSCs had potential for repairing T2DM rat skin wounds [90].

## 6. Challenges and Future Directions

### 6.1. Heterogeneity Barrier in Clinical Translation

Despite the therapeutic promise of MSCs in T2DM, their clinical translation is hindered by three major challenges. First, significant product heterogeneity arising from variations in cell isolation and culture conditions [106–109] underscores the critical need for rigorous quality control systems and standardized protocols to ensure batch‐to‐batch consistency [110–111]. This challenge is compounded by the marked heterogeneity of MSCs derived from different tissue sources. MSCs isolated from bone marrow, adipose tissue, umbilical cord, menstrual blood, and Wharton’s jelly exhibit distinct biological characteristics, immunogenicity profiles, and therapeutic efficacies [108, 111–114]. For instance, UC‐MSCs maintain stable low immunogenicity throughout passaging, whereas bone marrow‐ and adipose tissue‐derived MSCs may show higher immunogenicity [111]. Additionally, in vitro studies have demonstrated that menstrual blood‐derived MSCs (MenSCs) exhibit a superior capacity to differentiate into pancreatic β‐like cells compared with UC‐MSCs and dental pulp‐derived MSCs (DPSCs), whereas UC‐MSCs and BM‐MSCs showed similar effects in reducing blood glucose levels and preserving β cells [109, 114]. These source‐dependent functional differences underscore the need for careful selection of MSC sources in clinical applications. Beyond source‐related heterogeneity, variability in isolation protocols, culture conditions, and administration regimens across studies further contributes to inconsistencies in clinical outcomes and undermines reproducibility [109, 110]. Currently, there is no clear consensus on optimal MSC source, preparation methods, or administration protocols, and systematic investigations are urgently needed to establish standardized approaches [109, 114]. Moreover, the quality and functional potency of MSCs are profoundly influenced by donor‐specific factors, including age and underlying health status [111, 114–118]. Studies have shown that donor age can affect the proliferative capacity of bone marrow‐derived MSCs, and obesity‐related metabolic abnormalities may impair the migratory function of adipose‐derived MSCs [116]. Also, patient characteristics have been shown to influence treatment response, with older patients or those with long‐standing diabetes exhibiting less pronounced therapeutic benefits [117]. In parallel, the diabetic microenvironment itself poses a substantial barrier to MSC‐based therapy. This pathological milieu can compromise critical cellular functions, including viability, differentiation capacity, migratory function, and paracrine activity [114, 115]. Collectively, these findings highlight the need for individualized consideration in treatment design, taking into account the complex interaction between the specific factors of the donor and the patient themselves, which may shape the outcomes of MSC‐based therapy. These interconnected variables necessitate exacting optimization of isolation and culturing protocols, stringent quality control for safety, and the development of scalable, cost‐effective manufacturing systems [106–109]. Furthermore, maintaining patient health and MSC bioactivity during cell preparation demands meticulous quality control procedures to prevent contamination. Addressing these various heterogeneities, establishing unified standards for the production and application of MSCs, and systematically studying the differences in patients with T2DM are key steps in realizing the clinical application of MSCs in T2DM.

### 6.2. Progress and Challenges of Gene Editing in T2DM Therapy

Although gene‐editing tools, such as CRISPR/Cas9, offer new possibilities for treating T2DM, their safety and long‐term effects—including assessment of off‐target events and the risk of genomic instability—require thorough evaluation. Treating polygenic diseases like T2DM through genetic manipulation is considerably more challenging than targeting monogenic disorders, given that T2DM arises from a combination of genetic susceptibility, environmental factors, and metabolic comorbidities [119]. This complexity renders the precise targeting of a single gene insufficient, highlighting the necessity of developing more sophisticated multigene strategies.

**Figure 5:**
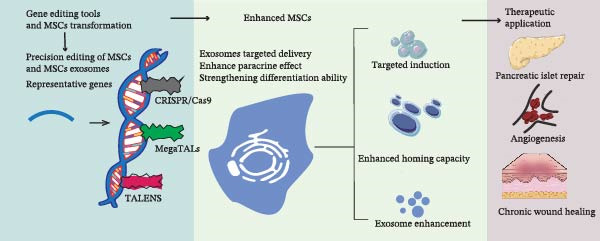
Gene‐editing tools enhance the therapeutic potential of MSCs. *Source:* This image is an original creation by the author.

This complexity makes precise targeting of a single gene insufficient, highlighting the need for more sophisticated multigene strategies. Despite these challenges, CRISPR/Cas9 has been extensively used to establish cell and animal models that help dissect the molecular mechanisms underlying T2DM. Examples include knockout models of MTNR1A/B, VEGFB, lepb, and GALNT14, as well as double‐knockout models of PPARα/γ in HepG2 and MIN6 cells, all of which have contributed to our understanding of insulin secretion, glucose homeostasis, and metabolic regulation [120, 121]. Beyond disease modeling, CRISPR‐based approaches have shown therapeutic promise. For instance, disrupting NRIP1 in adipocytes promotes browning and improves glucose tolerance following transplantation [122]. In addition, editing a lncRNA involved in a ceRNA network has been shown to restore normal GLUT4 and mTOR expressions [123]. Furthermore, macrophage‐specific Ntn1 silencing delivered via a nanoparticle‐based system ameliorated hyperglycemia in diabetic mice [124]. In addition, CRISPR has been explored as a tool to engineer probiotic strains such as *Akkermansia muciniphila* to enhance their survival and therapeutic efficacy in T2DM management. These examples illustrate the versatility of the CRISPR technology in both mechanistic exploration and therapeutic development. However, the application of this technology in MSCs is still at the exploratory stage. Ning et al. [125] studied MSC interaction with microvesicles and found that they induced the differentiation of MSCs into IPCs through miR‐181a‐5p/150‐5p, laying a theoretical foundation for enhancing the differentiation efficiency of MSCs via gene editing technologies. The study by Kim et al. [126] demonstrated that gene editing using the CRISPR–Cas9 system enabled the targeted induction of MSCs, providing an empirical foundation for optimizing stem cell‐based therapies. Similarly, Shimizu et al. [127] demonstrated how gene‐editing tools such as CRISPR/Cas9, megaTALs, and TALENs could be employed to significantly enhance the therapeutic potential of MSC‐derived EVs (MSC‐EVs) for patient‐centered wound healing by enabling precise genomic modifications in MSCs. Through gene editing, modulation, or overexpression of specific chemokines, the homing capacity of stem cells to specific targets can be significantly enhanced, their paracrine effects can be regulated, and the immunomodulatory capability of MSCs can be strengthened [128] (Figure 5).

Despite this progress, translating CRISPR‐based therapies into clinical practice requires overcoming several technical hurdles. Particularly for in vivo applications, where achieving tissue‐specific targeting while minimizing immune activation and off‐target edits is technically demanding [124, 129]. Off‐target effects, potential immunogenicity against Cas9 proteins, and the risk of genomic instability all warrant rigorous evaluation, and advances such as base editing and refined regulatory strategies are being explored to mitigate these risks [129]. Long‐term safety monitoring is essential to ascertain the presence of abnormal differentiation of stem cells in organs during treatment, not only to assess whether stem cells undergo abnormal differentiation in vivo that could lead to tumorigenesis but also to evaluate potential immune responses and other unforeseen adverse events associated with gene editing as this could cause tumorigenesis [130]. To enhance the therapeutic potential of MSCs in T2DM islets, chronic wounds, and vascular regeneration.

### 6.3. Application of Tissue Engineering Materials in the Differentiation of UC‐MSCs Into β‐Cells

**Figure 6:**
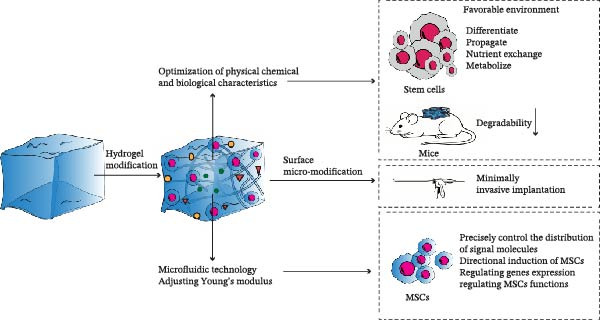
Modifying tissue engineering materials to improve biological properties and meet different application needs. *Source:* This image is an original creation by the author.

Tissue engineering strategies require precise integration of scaffold‐based physical and biochemical signals to guide cell fate decisions [131–134]. Although recent advances—such as photo‐crosslinkable matrices and microfluidic platforms—have improved the ability to regulate stem cell behavior, the field remains constrained by an incomplete understanding of how material properties influence the complex signaling networks between MSCs and their microenvironment [135, 136]. Studies have shown that material architecture and stiffness profoundly alter the morphology, proliferative capacity, and transcriptomic profiles of MSCs [137–140]. Lin et al. [140] demonstrated that in a stiffer matrix (with a modulus of ~18.29 kPa), the spreading area of MSCs was restricted, while significantly enhancing cell proliferation activity. RNA sequencing analysis revealed that, compared to the softer matrix, the stiffer matrix upregulated the expression of 111 genes and downregulated the expression of 44 genes in MSCs. These differentially expressed genes are primarily associated with the cell cycle, microtubule dynamics, and cell division. However, most of these findings derive from simplified in vitro systems, and further investigation is needed to fully elucidate the impact of materials on signaling interactions between stem cells and other cell types [141]. Moreover, the clinical translation of engineered scaffolds is hampered by inconsistencies in manufacturing, insufficient long‐term biocompatibility data, and a lack of long‐term clinical evidence [141, 142]. Future efforts must focus on precise mechanistic dissection of material–cell interactions in physiologically relevant disease models and the establishment of scalable, standards‐compliant manufacturing processes to realize the therapeutic potential of MSC–material constructs in T2DM and beyond (Figure 6).

## 7. Conclusions

Despite significant advances in stem cell‐based therapies for diabetes, their application in T2DM must account for the complexity of the disease—characterized not only by progressive β‐cell failure but also by a range of chronic complications (including cardiovascular, renal, and neuropathic disorders)—which collectively create an unfavorable microenvironment for graft cell survival. Key challenges remain, including issues related to scalable manufacturing, immune rejection, homing efficiency, and long‐term functional stability. Addressing these obstacles will require coordinated progress in stem cell expansion, enhancement of cellular resilience and reduction of immunogenicity through gene editing, and the development of biomaterial‐based microenvironments to support engraftment. By integrating these interdisciplinary approaches, future stem cell strategies hold promise not only for β‐cell replacement but also for achieving broader therapeutic benefits in T2DM and its associated complications.

## Author Contributions

**Jia Yang**: writing – original draft, methodology, investigation, data curation. **Yinlong Chen and Huanmei Jing**: methodology, investigation. **Fei Yu**: review and editing. **Chang Liu, Xiaolin Chen, and Jiaju Chu**: investigation. **Yang Liu**: writing – review and editing, conceptualization. **Wancong zhang**: writing – review and editing, project administration, conceptualization. **Shijie Tang**: project administration, conceptualization.

## Funding

This study was funded by the Basic and Applied Basic Research Foundation of Guangdong Province (Grants 2023A1515012343 and 2022A1515220099), the Li Ka Shing Foundation Cross‐Disciplinary Research Grant (Grants 2020LKSFG18B and 2020LKSFG02E), the Provincial Science and Technology Innovation Strategy Special Project Funding Program (Grants 200114165897946, 210714106901245, STKJ202209067, STKJ2023004, and STKJ2024066).

## Disclosure

All authors of this manuscript have read and approved the final version of the manuscript and confirm that the content of this review article has not been published elsewhere and is not under consideration by any other journal.

## Ethics Statement

The authors have nothing to report.

## Consent

The authors have nothing to report.

## Conflicts of Interest

The authors declare no conflicts of interest.

## Data Availability

Data sharing is not applicable to this article, as no datasets were generated or analyzed during the current study.
